# Changes in Prefrontal Gamma-Aminobutyric Acid and Perfusion After the Computerized Relaxation Training in Women With Psychological Distress: A Preliminary Report

**DOI:** 10.3389/fpsyg.2021.569113

**Published:** 2021-04-13

**Authors:** Eun Namgung, Jungyoon Kim, Hyeonseok Jeong, Jiyoung Ma, Gahae Hong, Ilhyang Kang, Jinsol Kim, Yoonji Joo, Rye Young Kim, In Kyoon Lyoo

**Affiliations:** ^1^Ewha Brain Institute, Ewha Womans University, Seoul, South Korea; ^2^Department of Brain and Cognitive Sciences, Ewha Womans University, Seoul, South Korea; ^3^Department of Radiology, College of Medicine, Incheon St. Mary's Hospital, The Catholic University of Korea, Seoul, South Korea; ^4^Department of Psychiatry, University of Utah School of Medicine, Salt Lake City, UT, United States; ^5^Graduate School of Pharmaceutical Sciences, Ewha Womans University, Seoul, South Korea

**Keywords:** stress, relaxation training, prefrontal cortex, gamma-aminobutyric acid, cerebral blood flow

## Abstract

Computerized relaxation training has been suggested as an effective and easily accessible intervention for individuals with psychological distress. To better elucidate the neural mechanism that underpins the effects of relaxation training, we investigated whether a 10-session computerized relaxation training program changed prefrontal gamma-aminobutyric acid (GABA) levels and cerebral blood flow (CBF) in women with psychological distress. We specifically focused on women since they were reported to be more vulnerable to develop stress-related disorders than men. Nineteen women with psychological distress but without a diagnosis of psychiatric disorders received the 10-day computerized relaxation training program that consisted of 30-min cognitive-relaxation training and 10-min breathing-relaxation training per day. At baseline and post-intervention, perceived stress levels, anxiety, fatigue, and sleep quality were assessed by self-report questionnaires. Brain magnetic resonance spectroscopy and arterial spin labeling scans were also performed before and after the intervention to evaluate GABA levels and relative CBF in the prefrontal region. Levels of perceived stress (*t* = 4.02, *P* < 0.001), anxiety (*z* = 2.33, *P* = 0.02), fatigue (*t* = 3.35, *P* = 0.004), and sleep quality (*t* = 4.14, *P* < 0.001) improved following 10 sessions of computerized relaxation training, resulting in a significant relief in composite scores of stress-related symptoms (*t* = −5.25, *P* < 0.001). The prefrontal GABA levels decreased (*t* = 2.53, *P* = 0.02), while relative CBF increased (*t* = −3.32, *P* = 0.004) after the intervention. In addition, a greater increase in relative prefrontal CBF was associated with better composite scores of stress-related symptoms following the intervention (*t* = 2.22, *P* = 0.04). The current findings suggest that computerized relaxation training may improve stress-related symptoms through modulating the prefrontal GABA levels and CBF in women with psychological distress.

## Introduction

Relaxation training has been reported to improve various stress-related symptoms (Bastani et al., 2005; Rausch et al., 2006). Keeping pace with recent technological advancement, several computerized relaxation training programs have been developed for mild to moderate psychological distress (Andrews et al., 2010; Mourad et al., 2016) with the advantages of accessibility, cost-effectiveness, and highly standardized contents (Mourad et al., 2016; Lyoo et al., 2019). The computerized relaxation training programs may be more relevant to the rapidly growing group of individuals with psychological distress who does not meet the diagnostic criteria of psychiatric disorders (Hidaka, 2012; Craske and Stein, 2016) as this group may prefer to use easily accessible programs rather than seeking psychological therapy or interventions. Despite its increasing usage and reported therapeutic efficacy in mild psychological distress (Poirier-Bisson et al., 2013; Kisely et al., 2015), the underlying neurobiological mechanisms of relaxation training need further investigations.

Changes in the prefrontal region may be related to the efficacy of relaxation training since dysfunctional top-down emotional regulation of the prefrontal region has been reported in psychological distress conditions (Hains and Arnsten, 2008; Ghosal et al., 2017). Specifically, previous studies suggested that the prefrontal gamma-aminobutyric acid (GABA) levels and cerebral blood flow (CBF) could be altered in individuals with psychological distress (Wang et al., 2005; Ghosal et al., 2017). The GABA levels and CBF were also recovered in relation to improved emotional regulation after cognitive-behavioral training in individuals with psychological distress (Guglietti et al., 2013; Tang et al., 2015, 2016; Ghosal et al., 2017), suggesting a close association with stress-related symptoms. As such, we assumed that the GABA levels and CBF might be neural correlates underlying the effects of relaxation training in psychological distress.

In the current study, we focused on the GABA levels since GABA has been known as a key inhibitory neurotransmitter in the brain that modulates interneuron activities and CBF, subsequently resulting in changes in stress reactivity (McQuail et al., 2015; Schür et al., 2016). In addition, we specifically applied the computerized relaxation training program on women with psychological distress since stress-related symptoms and disorders are more prevalent in women (Shansky et al., 2004; Cosgrove et al., 2007). We investigated whether the 10 daily sessions of computerized relaxation training program changed the prefrontal GABA and CBF as well as stress-related symptoms in women with psychological distress.

## Methods

### Participants

The participants were 19 young women aged between 19 and 45 years with subclinical psychological distress that lasted at least 1 month. Exclusion criteria were as follows: (1) symptoms met the criteria for current or lifetime axis one psychiatric disorders using the Structured Clinical Interview for Diagnostic and Statistical manual, fourth edition (SCID-IV) as assessed by an experienced clinician (First and Gibbon, 2004); (2) a history of traumatic brain injury with loss of consciousness; (3) presence of clinically significant medical or neurological disorders; or (4) any contraindications to brain magnetic resonance imaging (MRI). The study protocol was approved by the Institutional Review Board of Ewha W. University. Written informed consent was obtained from all the participants prior to enrollment.

### Interventions

The participants received 10 daily sessions of computerized relaxation training program during week days for 2 weeks. One daily session consisted of 30-min cognitive-relaxation and 10-min breathing-relaxation training. The clinician supervised and ensured that each of the participants attended to and completed all parts of the training. All training sessions were closely monitored and supervised by the clinician. The clinician asked every participant to self-report the level of concentration and write down any comments for each training session. The study was designed to exclude the data in which the self-rating for concentration on each session of the relaxation therapy was lower than 80%. Since all the ratings of the concentration provided by the participants for each treatment session were higher than 80%, indicating satisfactory compliance of the training, no data were excluded from the final analysis.

The 30-min cognitive-relaxation training is a computer-based interactive program that was developed and accordingly modified based on the standard cognitive behavioral therapy (Jonsbu et al., 2011, 2013). This program consists of the three subsections: brief education about causes and mechanisms of psychological distress, cognitive restructuring training for each automatic thinking, and relaxation training including progressive muscle relaxation and breathing techniques. The second 10-min breathing-relaxation training program instructs participants to clear their minds of thoughts with deep breathing and relaxation by focusing on their inhalation and exhalation.

### Outcome Measures: Stress-Related Symptoms

Composite scores of stress-related symptoms were assessed at baseline and post-intervention, including the following suggested as representative and interrelated domains of subclinical stress-related symptoms (Tang et al., 2007, 2015, 2016): perceived stress levels, anxiety, fatigue, and sleep quality. The Perceived Stress Scale (PSS-10), a 10-item self-report with a four-point scale, was used to measure subjective level of stress perception over the past month. The higher total scores of the PSS-10 indicate a higher level of perceived stress (Cohen et al., 1983; Roberti et al., 2006). The anxiety symptoms were assessed using the Beck Anxiety Inventory (BAI), a 21-item inventory with a three-point scale (Ulusoy et al., 1998). The total scores of the BAI were used for the level of anxiety, with a higher score indicating greater anxiety (Ulusoy et al., 1998). The Brief Fatigue-Inventory (BFI), a nine-item self-report with a 10-point scale, was used to assess excessive fatigue over the past week as well as fatigue severity over the past day (Yun et al., 2005). Finally, sleep quality was assessed using the Pittsburgh Sleep Quality Index (PSQI) (Buysse et al., 1989). The PSQI is a 10-item self-report scale with a three-point scale for measurement of global sleep quality over the past month (Buysse et al., 1989), with a higher score indicating more sleep-related problems (Buysse et al., 1989).

The standardized *z* scores of the PSS-10, BAI, BFI, and PSQI for baseline and post-intervention were calculated using the means and standard deviations (SD) of the respective baseline scale scores and reversed to indicate lower stress-related symptoms with a positive value. Composite scores for the stress-related symptoms were calculated by averaging *z* scores of the PSS-10, BAI, BFI, and PSQI at baseline and post-intervention, respectively. The composite scores encompassing perceived stress, anxiety, fatigue, and sleep problem were used to indicate subclinical stress-related symptoms that can generally manifest in various ways.

### Outcome Measures: Prefrontal GABA and Relative CBF

Prefrontal GABA levels and relative CBF were assessed using proton magnetic resonance spectroscopy (MRS) and arterial spin labeling (ASL) perfusion imaging, respectively, at baseline and after the completion of the 10-days training program. Brain MRI and MRS data were acquired using a 3.0-T Philips Magnetic Resonance scanner system (Philips Healthcare, Best, the Netherlands) equipped with a 32-channel head coil.

High-resolution three-dimensional T1-weighted images were obtained using a magnetization-prepared rapid gradient echo imaging sequence with the following parameters: echo time (TE) = 3.4 ms, repetition time (TR) = 7.4 ms, flip angle = 8°, number of excitation (NEX) = 1, field of view (FOV) = 224 × 224 mm^2^, voxel size = 1 × 1 mm^2^, slice thickness = 1 mm, and contiguous sagittal slices = 180. The T1-weighted images were used for spectroscopic voxel localization, tissue segmentation, and coregistration.

**Figure 1:**
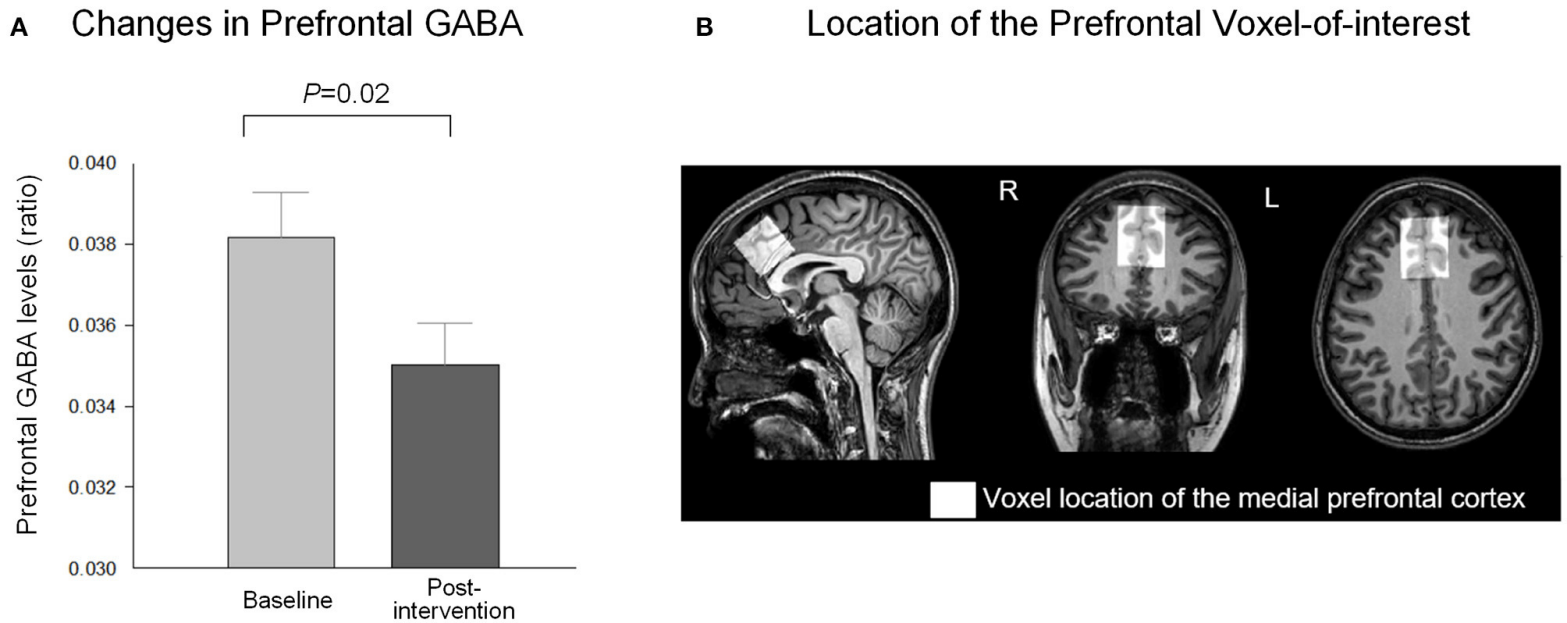
Changes in prefrontal GABA levels after the relaxation training. **(A)** Prefrontal GABA level decreased (*t* = 2.53, *P* = 0.02) following 10 daily sessions of the computerized relaxation training. **(B)** The location of the prefrontal region as voxel-of-interest of the study (3 × 3 × 3 cm^3^) is overlaid on the T1-weighted image of a representative study participant. GABA, gamma-aminobutyric acid.

Prefrontal GABA levels were measured using the Mescher–Garwood point-resolved spectroscopy (MEGA-PRESS) sequence as the simultaneous spectral editing technique. The voxel-of-interest (VOI, 3 × 3 × 3 cm^3^) was centered on the medial prefrontal region including mostly gray matter (GM) (Figure 1). The VOI was placed anterior to the genu of the corpus callosum and oriented along the bicommisural line in the sagittal plane as well as centered on the interhemispheric fissure in the coronal and axial planes of the T1-weighted images. The VOI at follow-up scan was placed based on the gyral pattern and location of the spectroscopic voxel based on the respective baseline prescription, in order to ensure the intrasubject reliability of voxel placement. The acquisition parameters for the MEGA-PRESS sequence were as follows: TE = 80 ms, TR = 2,000 ms, water suppression = multiply optimized insensitive suppression train (MOIST), pulse duration = 20 ms, number of signals averaged (NSA) = 2, phase cycle = 16, and dynamic scans = 160, with frequency stabilization. To reduce field inhomogeneities, second-order shim currents were adjusted with an automated shimming. The water suppression band is applied at a frequency of 4.68 ppm. Gaussian editing pulses are alternatively applied at 1.9 ppm (ON) and 1.5 ppm (OFF) in even- and odd-numbered acquisitions, respectively. The difference of the ON and OFF spectra provided a macromolecule-suppressed edited spectrum of GABA, detected at 3.0 ppm (Edden et al., 2014).

Prefrontal CBF was measured using pseudocontinuous ASL (pCASL) with a three-dimensional single-shot gradient and spin echo (3D-GRASE) sequence with the following parameters: TE = 12 ms, TR = 3,772 ms, FOV = 220 × 220 mm^2^, voxel size = 2.75 × 2.75 mm^2^, slice thickness = 6 mm, axial slices = 22, NSA = 1, post-labeling delay = 1,600 ms, and labeling duration = 1,800 ms, with background suppression. The mean equilibrium magnetization (M0) image was acquired using the same readout and TR but without labeling and background suppression. The labeling plane was positioned 96 mm inferior to the center of acquisition volume perpendicular to the internal carotid and basilar arteries, while avoiding branches and bifurcation (Aslan et al., 2010). We used the pCASL technique which has advantages of more direct and sole quantification of CBF in absolute units (ml blood/100 g tissue/min). Using the pCASL technique, water protons of the arterial blood are magnetically labeled and unlabeled in an alternating sequence at the labeling plane of the carotid vessels. The labeled and control images are generated after a short period of post-labeling delay in which the labeled or unlabeled blood protons travel upstream through the blood vessels and reach the field-of-view. Perfusion weighted images were then generated from the difference between labeled and unlabeled images (Zhou et al., 2015). In contrast, the blood-oxygen level-dependent (BOLD) functional magnetic resonance imaging (fMRI) measures changes in relaxation time of blood and tissue water secondary to changes in the ratio of deoxyhemoglobin and oxyhemoglobin (Ogawa et al., 1993). It is suggested that BOLD signal is a combination of the CBF, cerebral blood volume, and oxygen consumptions. To screen for the presence of neuroradiological abnormalities, fluid-attenuated inversion recovery axial images were also acquired with the following acquisition parameters: TE = shortest, 275–279 ms; TR = 4,800 ms; inverse time (TI) = 1,650 ms; FOV = 240 × 240 mm^2^; voxel size = 1 × 1 mm^2^; and slice thickness = 0.56 mm.

### MRS Data Processing

Quantification of GABA signal was performed using the Gannet 3.0, a MATLAB-based batch-processing toolkit for analyzing GABA-edited MRS spectra (Edden et al., 2014). Gannet processing consists of several modules: GannetLoad, GannetFit, GannetCoRegister, and GannetSegment. The time domain of proton MRS data was processed into a frequency-domain GABA-edited spectrum using the GannetLoad function that filters unsuppressed water and spectroscopy data with an exponential line broadening of 3 Hz. To maximize the quality of the edited spectrum, the prefrontal GABA levels were normalized using water signal as a reference compound, along with default parameters of spectral registration including frequency and phase corrections (Near et al., 2014). Subsequently, GABA signal from the spectral differences was fit to the edited GABA peak at 3 ppm using non-linear least-squares fitting from GannetFit, with *N*-acetyl-l-aspartate (NAA) as an internal standard for GABA quantification. The estimated GABA concentration was then registered to the respective T1-weighted image using GannetCoRegister, creating the binary mask of the VOI that uses the same geometry as the T1-weighted image. The VOI of each T1-weighted image was segmented according to fractions of GM, white matter (WM), and CSF using GannetSegment implemented by statistical parametric mapping (SPM), as to provide tissue-corrected GABA quantification. Adequate spectra were confirmed by visual inspection as well as using fitting errors shown in the Gannet toolkit. One participant with the spectrum with normalized residual fitting error above 10 at pre-intervention and the intervention-related changes in GABA/NAA ratio above 2 SD of the group mean was excluded from further analysis of prefrontal GABA levels. The current study used the GABA/NAA ratio as the measure of GABA level throughout all analyses.

### ASL Data Processing

All volumes of the ASL were first realigned to minimize motion-related artifacts. The absolute CBF maps of each participant were reconstructed after pairwise subtraction of control and label images and calibration in reference to the M0 image. The FMRIB Software Library (FSL) tools (https://fsl.fmrib.ox.ac.uk/fsl) were used to process ASL images as well as to quantify absolute CBF in the prefrontal region and whole-brain GM. The absolute CBF maps were first denoised in reference to binary mask of M0 images that were separated and skull-stripped from the four-dimensional ASL images.

The CBF values from the prefrontal VOI were extracted in ASL native space with the following process. Individual denoised CBF maps in ASL space were coregistered to the corresponding skull-stripped T1-weighted images using linear transformation. In parallel, high-resolution T1-weighted images were segmented using the FMRIB's Automated Segmentation Tool of FSL (Zhang et al., 2001) to create GM partial volume images. Subject-level GM masks were created with a threshold of 0.8 and used to extract resting CBF predominantly within GM of the brain. Individual GM binary masks in T1 space were then registered to the corresponding denoised ASL images using the inverse transformation matrix of the abovementioned registration of ASL to T1 images in the native space. The prefrontal VOI for measurement of GABA levels in T1 space was also registered to the corresponding denoised ASL images using the inverse transformation matrix of the abovementioned registration. Absolute CBF values within the prefrontal VOI were normalized as a ratio to the mean absolute CBF value of the whole-brain GM in order to account for the subject-level differences. The prefrontal CBF to global CBF ratios were then used in the final analysis.

### Statistical Analysis

Changes in stress-related symptoms after the relaxation training were evaluated using paired *t* tests and a Wilcoxon signed-rank test for variables with or without a normal distribution, respectively. In addition, changes in prefrontal GABA levels and relative CBF were also assessed using paired *t* tests. We also examined whether changes in prefrontal GABA and relative CBF might be associated with the improvement of stress-related symptom after the 10-session intervention, using regression models including the following variables: changes in prefrontal GABA level or relative CBF as an independent variable and composite stress-related symptom scores after the intervention as a dependent variable of perceived stress level, anxiety, fatigue, and sleep quality. Two-tailed significance of *P* < 0.05 was considered to be statistically significant. Statistical tests were performed using Stata SE version 13.1 (StataCorp LP, College Station, Texas).

## Results

### Participant Characteristics

Nineteen women (mean ± SD, 28.0 ± 2.76 years) with subclinical psychological distress reported perceived stress level ranging from minimal to severe degrees (mean ± SD, 19.8 ± 8.17, range 7–33) (Cohen et al., 1983; Roberti et al., 2006), despite that all the participants did not meet the criteria for current or lifetime axis 1 psychiatric disorders according to the SCID-IV, including stress-related psychiatric disorders. In contrast, anxiety was minimal to mild degrees (mean ± SD, 5.42 ± 4.62, range 0–15) as well as fatigue (mean ± SD, 4.05 ± 2.28, range 0.44–8) and poor sleep quality (mean ± SD, 5.47 ± 1.98, range 3–10) were minimal to moderate degrees (Buysse et al., 1989; Ulusoy et al., 1998; Yun et al., 2005). Education levels were 18.3 ± 1.50 years.

### Effects of Relaxation Training on Stress-Related Symptoms and Prefrontal GABA and CBF

The participants showed significant improvement in the composite scores of stress-related symptoms after receiving 10 daily sessions of the computerized relaxation training program (*t* = −5.25, *P* < 0.001, *df* = 18) (Table 1). Perceived stress levels (*t* = 4.02, *P* < 0.001, *df* = 18), anxiety (*z* = 2.33, *P* = 0.02, *df* = 18), fatigue (*t* = 3.35, *P* = 0.004, *df* = 18), and sleep quality (*t* = 4.14, *P* < 0.001, *df* = 18) all showed improvement following the intervention (Table 1).

**Table 1:** Changes in clinical outcome measures after the relaxation training.

	**Baseline (*n* = 19)**		**Post-intervention (*n* = 19)**		***P***
	**Mean**	**SD**	**Mean**	**SD**	
**Stress-related symptoms**					
PSS-10, total scores	19.8	8.17	15.6	7.52	<0.001
BAI, total scores	5.42	4.62	3.58	3.52	0.02
BFI, total scores	4.05	2.28	2.92	1.63	0.004
PSQI, total scores	5.47	1.98	4.47	1.74	<0.001
Composite *z* scores^*^	0.00	0.79	0.48	0.58	<0.001

*Stress-related symptoms were compared between the baseline and post-intervention using paired t tests or a Wilcoxon signed-rank test in the variables with or without normal distribution, respectively*.

*BAI, Beck Anxiety Inventory; BFI, Brief Fatigue Inventory; PSS-10, Perceived Stress Scale−10; PSQI, Pittsburgh Sleep Quality Index; SD, standard deviation*.

* *The composite z scores of the stress-related symptoms are generated from the Perceived Stress Scale-10, Beck Anxiety Inventory, Brief Fatigue Inventory, and Pittsburgh Sleep Quality Index. Positive values in the composite z scores indicate improved symptom scores after the intervention*.

**Figure 2:**
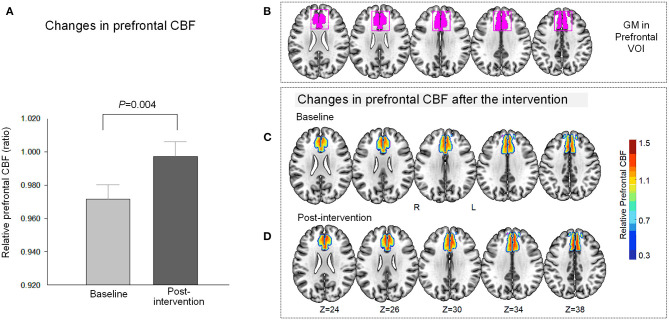
Changes in relative prefrontal CBF after the relaxation training. **(A)** The relative CBF within the medial prefrontal region increased (*t* = −3.32, *P* = 0.004) in relation to the 10 daily sessions of the computerized relaxation training program. **(B)** Anatomical localization of the prefrontal VOI and the gray matter within the VOI of the study are presented as violet-colored box and violet-colored binary map on the brain template, respectively. The relative CBF map within the prefrontal VOI was overlaid on the brain template for at baseline **(C)** and after the intervention **(D)**. The red color indicates higher relative CBF, while the blue color indicates lower relative CBF as indicated in the vertical color bar. CBF, cerebral blood flow; GM, gray matter; VOI, voxel-of-interest.

The prefrontal GABA levels decreased (*t* = 2.53, *P* = 0.02, *df* = 17) after the 10-day relaxation training program (Figure 1), while the relative prefrontal CBF increased (*t* = −3.32, *P* = 0.004, *df* = 18) in relation to the 10 daily sessions of the computerized relaxation training (Figure 2).

**Figure 3:**
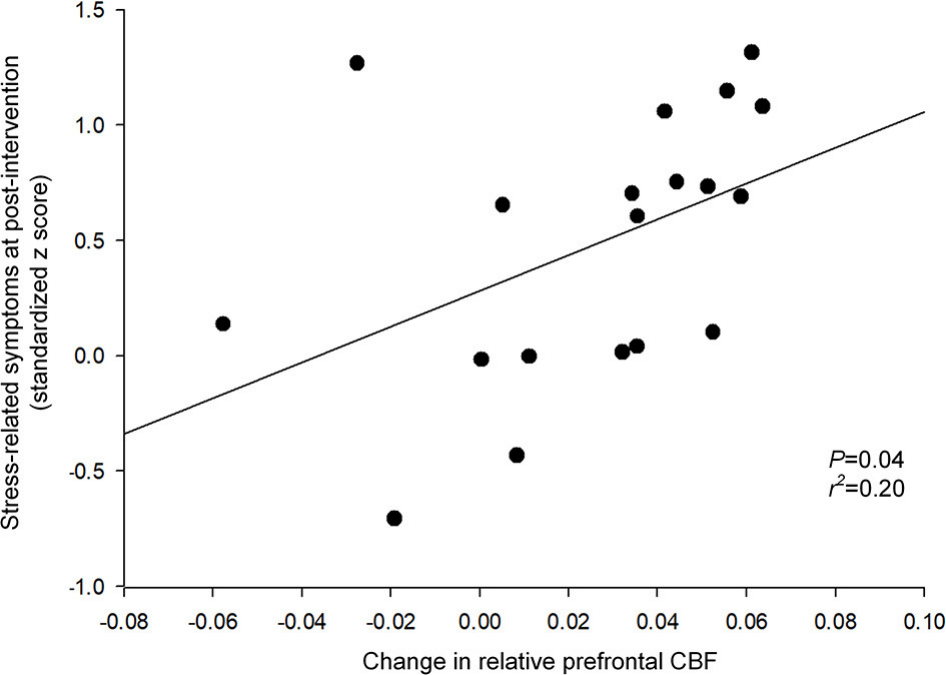
Relationship between changes in relative prefrontal CBF and stress-related symptoms at post-intervention. A greater magnitude of relative CBF increase in the prefrontal region was associated with better stress-related composite scores following the intervention (*t* = 2.22, *P* = 0.04). The relationship was tested using a robust regression model that included the following variables: changes in relative prefrontal CBF as an independent variable, and the stress-related symptoms at post-intervention as a dependent variable. The regression line is indicated in a solid line. CBF, cerebral blood flow.

A greater magnitude of relative CBF increase in the prefrontal region was associated with better stress-related composite scores following the intervention (*t* = 2.22, *P* = 0.04, *df* = 18) (Figure 3). In contrast, a decrease in the prefrontal GABA level was not associated with the composite stress-related scores at post-intervention (*t* = 0.54, *P* = 0.60, *df* = 17). Changes in the prefrontal GABA level and prefrontal CBF after the relaxation training were not associated (*t* = 1.53, *P* = 0.15, *df* = 17).

## Discussion

In the current study, we report that computerized relaxation training may induce GABA level and CBF changes in the prefrontal region of the brain in women with psychological distress. These finding may suggest that prefrontal GABA and CBF are neurobiologically relevant correlates that underlie the effects of relaxation training. To the best of our knowledge, the present study is the first to investigate prefrontal GABA and perfusion changes simultaneously using multimodal neuroimaging in relation to the relaxation training. We cautiously infer that decreased prefrontal GABA levels, as induced by the relaxation training, may increase the prefrontal perfusion. Consequently, changes in the prefrontal GABA levels and perfusion may have enhanced the cognitive control of psychological distress as well as improve stress-related symptoms.

Recent evidence highlights the clinical significance of GABA inhibitory control with regard to stress-related symptoms as well as extrasynaptic GABA receptors as a key modulator of major cortical neuronal activities (McQuail et al., 2015; Schür et al., 2016). Prefrontal neurotransmission through GABAergic receptors may undergo dynamic alterations in response to psychological distress (Hasler et al., 2010; Schür et al., 2016). These alterations in prefrontal GABAergic inhibition were suggested to play a putative role in subsequent development (Hasler et al., 2007, 2010) or recovery (Sanacora et al., 2006; Bhagwagar et al., 2008) of stress-related symptoms. Lower inhibitory GABA levels may have activated the prefrontal regions critically involved in stress and sleep regulation (Depue et al., 2007; Ghosal et al., 2017). This is supported by the previously reported negative associations between GABA levels and activation within the prefrontal regions (Michels et al., 2012; Hu et al., 2013).

Clinical improvement in perceived stress, anxiety, fatigue, and sleep quality found in this study is in alignment with previous reports on the effects of relaxation training on stress-related symptoms (Jonsbu et al., 2013; Van Beek et al., 2013) and sleep disturbances (Morin, 2004; Cheng and Dizon, 2012) that highly co-occur given their shared neurobiological mechanisms (Voelker, 2004). The current finding also provides supportive evidence regarding the crucial involvement of increased prefrontal activity in stress regulation. Increased prefrontal activity has been reported in associations with improved psychological distress such as anxiety and fatigue after the short-term integrative body–mind training (Tang et al., 2007, 2015) and meditation (Davidson et al., 2003). Moreover, increased prefrontal activation was related to responsiveness to CBT for stress-related psychiatric disorders (Maslowsky et al., 2010; Goldin et al., 2013).

The medial prefrontal cortex where we measured the GABA levels and CBF in this study is a highly connected hub region within the default mode network (Papez, 1995; Rothbart et al., 2011). This region is known to regulate arousal and sleep–wake cycles that underlie a broad range of stress-related conditions through top-down control of the limbic structures as well as modulations of functional balance between brain networks (Lyoo et al., 2012; Ha et al., 2019). Increased perfusion in the medial prefrontal region as a major hub of the default mode network may suggest a strengthened top-down cognitive control of the emotional distress through indirect inhibitory connections mediated by GABA (Hariri et al., 2003). This is also corroborated by the previous findings on enhanced connectivity and network efficiency of the prefrontal cortex (Tang et al., 2013, 2016) as well as increased prefrontal and decreased limbic activities (Phan et al., 2005; Wager et al., 2008) in associations with better stress regulation after interventions. In addition, previously reported negative associations between prefrontal GABA levels and activation (Michels et al., 2012; Hu et al., 2013; Tang et al., 2013) suggest that prefrontal GABAergic receptors and inhibitory interneurons may undergo the intervention-related changes. These changes were also associated with the modification in binding affinity (Brickley and Mody, 2012; McQuail et al., 2015) that is potentially related to increased prefrontal CBF and improved stress-related symptoms by modulating the activation–inhibition network and sleep–wake cycle (Muzur et al., 2002; Wager et al., 2008).

The following limitations should be considered in interpreting the results of the current study. Given higher prevalence of female depression, the study participants included only women with psychological distress to minimize potential cofounding effects due to sex differences. Future larger studies including both women and men with chronic stress-related conditions are needed to generalize the current findings. Moreover, studies with the control group are warranted to test potential placebo effects of the relaxation training on the prefrontal GABA and CBF underlying improved stress regulation as well as between-group differences in brain and clinical measures. We used a relatively lower resolution of 3 × 3 × 3 cm^3^ to precisely measure the GABA levels, as being separated from Glx level, using MEGA-PRESS sequence, rather than measuring Glx levels with higher resolution sequence. The prefrontal cortex was predefined as the region-of-interest, considering its critical involvement in emotional regulation of psychological stress. Further study that assesses the effects of relaxation training on subclinical stress needs to use depression scales and the whole-brain approach in neuroimaging to broaden perspectives on stress-related brain mechanisms.

## Data Availability Statement

The raw data supporting the conclusions of this article will be made available by the authors, without undue reservation.

## Ethics Statement

The studies involving human participants were reviewed and approved by Institutional Review Board of the Ewha Womans University. The patients/participants provided their written informed consent to participate in this study.

## Author Contributions

JuK and IL designed the study and obtained funding. JM, GH, IK, JiK, YJ, and RK collected the data. EN and HJ conducted the analysis. EN, JuK, HJ, and IL interpreted the results and drafted the manuscript. All authors revised and reviewed the manuscript.

## Conflict of Interest

The authors declare that the research was conducted in the absence of any commercial or financial relationships that could be construed as a potential conflict of interest.

## References

[B1] Andrews G. Cuijpers P. Craske M. G. McEvoy P. Titov N. (2010). Computer therapy for the anxiety and depressive disorders is effective, acceptable and practical health care: a meta-analysis. PLoS ONE 5:e13196. 10.1371/journal.pone.0013196 20967242 PMC2954140

[B2] Aslan S. Xu F. Wang P. L. Uh J. Yezhuvath U. S. Van Osch M.. (2010). Estimation of labeling efficiency in pseudocontinuous arterial spin labeling. Magn. Reson. Med. 63, 765–771. 10.1002/mrm.22245 20187183 PMC2922009

[B3] Bastani F. Hidarnia A. Kazemnejad A. Vafaei M. Kashanian M. (2005). A randomized controlled trial of the effects of applied relaxation training on reducing anxiety and perceived stress in pregnant women. J. Midwifery Women Health 50, e36–e40. 10.1016/j.jmwh.2004.11.008 15973255

[B4] Bhagwagar Z. Wylezinska M. Jezzard P. Evans J. Boorman E. Matthews P. M.. (2008). Low GABA concentrations in occipital cortex and anterior cingulate cortex in medication-free, recovered depressed patients. Int. J. Neuropsychopharmacol. 11, 255–260. 10.1017/S1461145707007924 17625025

[B5] Brickley S. G. Mody I. (2012). Extrasynaptic GABAA receptors: their function in the CNS and implications for disease. Neuron 73, 23–34. 10.1016/j.neuron.2011.12.012 22243744 PMC3399243

[B6] Buysse D. J. Reynolds I. I. I. C. F. Monk T. H. Berman S. R. Kupfer D. J. (1989). The Pittsburgh Sleep Quality Index: a new instrument for psychiatric practice and research. Psychiatry Res. 28, 193–213. 10.1016/0165-1781(89)90047-4 2748771

[B7] Cheng S. K. Dizon J. (2012). Computerised cognitive behavioural therapy for insomnia: a systematic review and meta-analysis. Psychother. Psychosom. 81, 206–216. 10.1159/000335379 22585048

[B8] Cohen S. Kamarck T. Mermelstein R. (1983). A global measure of perceived stress. J. Health Soc. Behav. 24, 385–396. 10.2307/2136404 6668417

[B9] Cosgrove K. P. Mazure C. M. Staley J. K. (2007). Evolving knowledge of sex differences in brain structure, function, and chemistry. Biol. Psychiatry 62, 847–855. 10.1016/j.biopsych.2007.03.001 17544382 PMC2711771

[B10] Craske M. G. Stein M. B. (2016). Anxiety. Lancet 388, 3048–3059. 10.1016/S0140-6736(16)30381-6 27349358

[B11] Davidson R. J. Kabat-Zinn J. Schumacher J. Rosenkranz M. Muller D. Santorelli S. F.. (2003). Alterations in brain and immune function produced by mindfulness meditation. Psychosom. Med. 65, 564–570. 10.1097/01.PSY.0000077505.67574.E3 12883106

[B12] Depue B. E. Curran T. Banich M. T. (2007). Prefrontal regions orchestrate suppression of emotional memories via a two-phase process. Science 317, 215–219. 10.1126/science.1139560 17626877

[B13] Edden R. A. Puts N. A. Harris A. D. Barker P. B. Evans C. J. (2014). Gannet: a batch-processing tool for the quantitative analysis of gamma-aminobutyric acid–edited MR spectroscopy spectra. J. Magn. Reson. Imaging 40, 1445–1452. 10.1002/jmri.24478 25548816 PMC4280680

[B14] First M. B. Gibbon M. (2004). The Structured Clinical Interview for DSM-IV Axis I Disorders (SCID-I) and the Structured Clinical Interview for DSM-IV Axis II Disorders (SCID-II). Hoboken: Wiley.

[B15] Ghosal S. Hare B. D. Duman R. S. (2017). Prefrontal cortex GABAergic deficits and circuit dysfunction in the pathophysiology and treatment of chronic stress and depression. Curr. Opin. Behav. Sci. 14, 1–8. 10.1016/j.cobeha.2016.09.012 27812532 PMC5086803

[B16] Goldin P. R. Ziv M. Jazaieri H. Hahn K. Heimberg R. Gross J. J. (2013). Impact of cognitive behavioral therapy for social anxiety disorder on the neural dynamics of cognitive reappraisal of negative self-beliefs: randomized clinical trial. JAMA Psychiatry 70, 1048–1056. 10.1001/jamapsychiatry.2013.234 23945981 PMC4141477

[B17] Guglietti C. L. Daskalakis Z. J. Radhu N. Fitzgerald P. B. Ritvo P. (2013). Meditation-related increases in GABAB modulated cortical inhibition. Brain Stimul. 6, 397–402. 10.1016/j.brs.2012.08.005 23022436

[B18] Ha E. Hong H. Kim T. D. Hong G. Lee S. Kim S.. (2019). Efficacy of Polygonatum sibiricum on mild insomnia: a randomized placebo-controlled trial. Nutrients 11:1719. 10.3390/nu11081719 31349690 PMC6723095

[B19] Hains A. B. Arnsten A. F. (2008). Molecular mechanisms of stress-induced prefrontal cortical impairment: implications for mental illness. Learn. Mem. 15, 551–564. 10.1101/lm.921708 18685145

[B20] Hariri A. R. Mattay V. S. Tessitore A. Fera F. Weinberger D. R. (2003). Neocortical modulation of the amygdala response to fearful stimuli. Biol. Psychiatry 53, 494–501. 10.1016/S0006-3223(02)01786-9 12644354

[B21] Hasler G. van der Veen J. W. Grillon C. Drevets W. C. Shen J. (2010). Effect of acute psychological stress on prefrontal GABA concentration determined by proton magnetic resonance spectroscopy. Am. J. Psychiatry 167, 1226–1231. 10.1176/appi.ajp.2010.09070994 20634372 PMC3107037

[B22] Hasler G. van der Veen J. W. Tumonis T. Meyers N. Shen J. Drevets W. C. (2007). Reduced prefrontal glutamate/glutamine and γ-aminobutyric acid levels in major depression determined using proton magnetic resonance spectroscopy. Arch. Gen. Psychiatry 64, 193–200. 10.1001/archpsyc.64.2.193 17283286

[B23] Hidaka B. H. (2012). Depression as a disease of modernity: explanations for increasing prevalence. J. Affect. Disord. 140, 205–214. 10.1016/j.jad.2011.12.036 22244375 PMC3330161

[B24] Hu Y. Chen X. Gu H. Yang Y. (2013). Resting-state glutamate and GABA concentrations predict task-induced deactivation in the default mode network. J. Neurosci 33, 18566–18573. 10.1523/JNEUROSCI.1973-13.2013 24259578 PMC3834059

[B25] Jonsbu E. Dammen T. Morken G. Moum T. Martinsen E. W. (2011). Short-term cognitive behavioral therapy for non-cardiac chest pain and benign palpitations: a randomized controlled trial. J. Psychosomat. Res. 70, 117–123. 10.1016/j.jpsychores.2010.09.013 21262413

[B26] Jonsbu E. Martinsen E. W. Morken G. Moum T. Dammen T. (2013). Change and impact of illness perceptions among patients with non-cardiac chest pain or benign palpitations following three sessions of CBT. Behav. Cognit. Psychother. 41, 398–407. 10.1017/S1352465813000179 23507293

[B27] Kisely S. R. Campbell L. A. Yelland M. J. Paydar A. (2015). Psychological interventions for symptomatic management of non-specific chest pain in patients with normal coronary anatomy. Cochrane Database Syst. Rev. 2015:CD004101. 10.1002/14651858.CD004101.pub5 26123045 PMC6599861

[B28] Lyoo I. K. Kim J. Y. Kim J. E. (2019). Efficacy of computerized cognitive behavioral therapy in individuals with non-cardiac checst discomfort: review and suggestions for a new protocol. Korean. J. Biol. Psych. 26, 1–7. 10.0000/kjbp.2019.26.1.1

[B29] Lyoo I. K. Yoon S. Jacobson A. M. Hwang J. Musen G. Kim J. E.. (2012). Prefrontal cortical deficits in type 1 diabetes mellitus: brain correlates of comorbid depression. Arch. Gen. Psychiatry 69, 1267–1276. 10.1001/archgenpsychiatry.2012.543 23090665 PMC4681445

[B30] Maslowsky J. Mogg K. Bradley B. P. McClure-Tone E. Ernst M. Pine D. S.. (2010). A preliminary investigation of neural correlates of treatment in adolescents with generalized anxiety disorder. J.Child Adolesc. Psychopharmacol. 20, 105–111. 10.1089/cap.2009.0049 20415605 PMC2865364

[B31] McQuail J. A. Frazier C. J. Bizon J. L. (2015). Molecular aspects of age-related cognitive decline: the role of GABA signaling. Trends Mol. Med. 21, 450–460. 10.1016/j.molmed.2015.05.002 26070271 PMC4500156

[B32] Michels L. Martin E. Klaver P. Edden R. Zelaya F. Lythgoe D. J.. (2012). Frontal GABA levels change during working memory. PLoS ONE 7:e31933. 10.1371/journal.pone.0031933 22485128 PMC3317667

[B33] Morin C. M. (2004). Cognitive-behavioral approaches to the treatment of insomnia. J. Clin. Psychiatry 65, 33–40. 15575803

[B34] Mourad G. Strömberg A. Jonsbu E. Gustafsson M. Johansson P. Jaarsma T. (2016). Guided Internet-delivered cognitive behavioural therapy in patients with non-cardiac chest pain–a pilot randomized controlled study. Trials 17:352. 10.1186/s13063-016-1491-1 27456689 PMC4960843

[B35] Muzur A. Pace-Schott E. F. Hobson J. A. (2002). The prefrontal cortex in sleep. Trends Cogn. Sci 6, 475–481. 10.1016/S1364-6613(02)01992-7 12457899

[B36] Near J. Ho Y.-C. L. Sandberg K. Kumaragamage C. Blicher J. U. (2014). Long-term reproducibility of GABA magnetic resonance spectroscopy. Neuroimage 99, 191–196. 10.1016/j.neuroimage.2014.05.059 24875142

[B37] Ogawa S. Lee T. Barrere B. (1993). The sensitivity of magnetic resonance image signals of a rat brain to changes in the cerebral venous blood oxygenation. Magn. Reson. Med. 29, 205–210. 10.1002/mrm.1910290208 8429784

[B38] Papez J. W. (1995). A proposed mechanism of emotion. 1937. J. Neuropsychiatry Clin. Neurosci. 7, 103–112. 10.1176/jnp.7.1.103 7711480

[B39] Phan K. L. Fitzgerald D. A. Nathan P. J. Moore G. J. Uhde T. W. Tancer M. E. (2005). Neural substrates for voluntary suppression of negative affect: a functional magnetic resonance imaging study. Biol. Psychiatry 5, 210–219. 10.1016/j.biopsych.2004.10.030 15691521

[B40] Poirier-Bisson J. Marchand A. Pelland M.-E. Lessard M.-J. Dupuis G. Fleet R.. (2013). Incremental cost-effectiveness of pharmacotherapy and two brief cognitive-behavioral therapies compared with usual care for panic disorder and noncardiac chest pain. J. Nerv. Ment. Dis. 201, 753–759. 10.1097/NMD.0b013e3182a2127d 23995030

[B41] Rausch S. M. Gramling S. E. Auerbach S. M. (2006). Effects of a single session of large-group meditation and progressive muscle relaxation training on stress reduction, reactivity, and recovery. Int. J. Stress. Manage 13:273. 10.1037/1072-5245.13.3.273

[B42] Roberti J. W. Harrington L. N. Storch E. A. (2006). Further psychometric support for the 10-item version of the perceived stress scale. J. Coll. Couns. 9, 135–147. 10.1002/j.2161-1882.2006.tb00100.x

[B43] Rothbart M. K. Sheese B. E. Rueda M. R. Posner M. I. (2011). Developing mechanisms of self-regulation in early life. Emot. Rev. 3, 207–213. 10.1177/1754073910387943 21892360 PMC3164871

[B44] Sanacora G. Fenton L. R. Fasula M. K. Rothman D. L. Levin Y. Krystal J. H.. (2006). Cortical γ-aminobutyric acid concentrations in depressed patients receiving cognitive behavioral therapy. Biol. Psychiatry 59, 284–286. 10.1016/j.biopsych.2005.07.015 16139814

[B45] Schür R. R. Draisma L. W. Wijnen J. P. Boks M. P. Koevoets M. G. Joëls M.. (2016). Brain GABA levels across psychiatric disorders: a systematic literature review and meta-analysis of 1H-MRS studies. Hum. Brain Mapp. 37, 3337–3352. 10.1002/hbm.23244 27145016 PMC6867515

[B46] Shansky R. Glavis-Bloom C. Lerman D. McRae P. Benson C. Miller K.. (2004). Estrogen mediates sex differences in stress-induced prefrontal cortex dysfunction. Mol. Psychiatr. 9:531. 10.1038/sj.mp.4001435 14569273

[B47] Tang Y.-Y. Tang R. Posner M. I. (2016). Mindfulness meditation improves emotion regulation and reduces drug abuse. Drug Alcohol Depend. 163, S13–S18. 10.1016/j.drugalcdep.2015.11.041 27306725

[B48] Tang Y. Y. Lu Q. Feng H. Tang R. Posner M. I. (2015). Short-term meditation increases blood flow in anterior cingulate cortex and insula. Front. Psychol. 6:212. 10.3389/fpsyg.2015.00212 25767459 PMC4341506

[B49] Tang Y. Y. Ma Y. Wang J. Fan Y. Feng S. Lu Q.. (2007). Short-term meditation training improves attention and self-regulation. Proc. Natl. Acad. Sci. U.S.A. 104, 17152–17156. 10.1073/pnas.0707678104 17940025 PMC2040428

[B50] Tang Y. Y. Tang R. Posner M. I. (2013). Brief meditation training induces smoking reduction. Proc. Natl. Acad. Sci. U.S.A. 110, 13971–13975. 10.1073/pnas.1311887110 23918376 PMC3752264

[B51] Ulusoy M. Sahin N. H. Erkmen H. (1998). The Beck anxiety inventory: psychometric properties. J. Cogn. Psychother. 12, 163–172.

[B52] Van Beek M. Oude Voshaar R. Beek A. Van Zijderveld G. Visser S. Speckens A.. (2013). A brief cognitive-behavioral intervention for treating depression and panic disorder in patients with noncardiac chest pain: a 24-week randomized controlled trial. Depress. Anxiety 30, 670–678. 10.1002/da.22106 23625592

[B53] Voelker R. (2004). Stress, sleep loss, and substance abuse create potent recipe for college depression. Jama 291, 2177–2179. 10.1001/jama.291.18.2177 15138228

[B54] Wager T. D. Davidson M. L. Hughes B. L. Lindquist M. A. Ochsner K. N. (2008). Prefrontal-subcortical pathways mediating successful emotion regulation. Neuron 59, 1037–1050. 10.1016/j.neuron.2008.09.006 18817740 PMC2742320

[B55] Wang J. Rao H. Wetmore G. S. Furlan P. M. Korczykowski M. Dinges D. F.. (2005). Perfusion functional MRI reveals cerebral blood flow pattern under psychological stress. Proc. Natl. Acad. Sci. U.S.A. 102, 17804–17809. 10.1073/pnas.0503082102 16306271 PMC1292988

[B56] Yun Y. H. Wang X. S. Lee J. S. Roh J. W. Lee C. G. Lee W. S.. (2005). Validation study of the korean version of the brief fatigue inventory. J. Pain Symptom Manag. 29, 165–172. 10.1016/j.jpainsymman.2004.04.013 15733808

[B57] Zhang Y. Brady M. Smith S. (2001). Segmentation of brain MR images through a hidden Markov random field model and the expectation-maximization algorithm. IEEE Trans. Med. Imaging 20, 45–57. 10.1109/42.906424 11293691

[B58] Zhou Y. Rodgers Z. B. Kuo A. H. (2015). Cerebrovascular reactivity measured with arterial spin labeling and blood oxygen level dependent techniques. Magn. Reson. Imaging. 33, 566–567. 10.1016/j.mri.2015.02.018 25708263 PMC4426232

